# Gamma entrainment frequency affects mood, memory and cognition: an exploratory pilot study

**DOI:** 10.1186/s40708-020-00119-9

**Published:** 2020-11-23

**Authors:** Ryan L. S. Sharpe, Mufti Mahmud, M. Shamim Kaiser, Jianhui Chen

**Affiliations:** 1 Department of Computer Science, Nottingham Trent University, Nottingham, NG11 8NS UK; 2 Medical Technology Innovation Facility, Nottingham Trent University, Nottingham, NG11 8NS UK; 3 Institute of Information Engineering, Jahangirnagar University, Savar, 1342 Dhaka, Bangladesh; 4 Beijing Key Laboratory of MRI and Brain Informatics, Beijing University of Technology, Beijing, 100124 China; 5 Beijing International Collaboration Base on Brain Informatics and Wisdom Services, Beijing, China

**Keywords:** Gamma frequency, 40 Hz, Cognition improvements, Memory improvements, Mood improvements

## Abstract

Here we provide evidence with an exploratory pilot study that through the use of a Gamma 40 Hz entrainment frequency, mood, memory and cognition can be improved with respect to a 9-participant cohort. Participants constituted towards three binaural entrainment frequency groups: the 40 Hz, 25 Hz and 100 Hz. Participants attended a total of eight entrainment frequency sessions twice over the duration of a 4-week period. Additionally, participants were assessed based on their cognitive abilities, mood as well as memory, where the cognitive and memory assessments occurred before and after a 5-min binaural beat stimulation. The mood assessment scores were collected from sessions 1, 4 and 8, respectively. With respect to the Gamma 40 Hz entrainment frequency population, we observed a mean improvement in cognitive scores, elevating from 75% average to 85% average upon conclusion of the experimentation at weak statistical significance ($\alpha$ = 0.10, *p* = 0.076). Similarly, memory score improvements at a greater significance ($\alpha$ = 0.05, *p* = 0.0027) were noted, elevating from an average of 87% to 95%. In pertinence to the mood scores, a negative correlation across all populations were noted, inferring an overall increase in mood due to lower scores correlating with elevated mood. Finally, correlation analysis revealed a stronger *R*$^2$ value (0.9838) within the 40 Hz group between sessions as well as mood score when compared across the entire frequency group cohort.

## Introduction

**Fig. 1:**
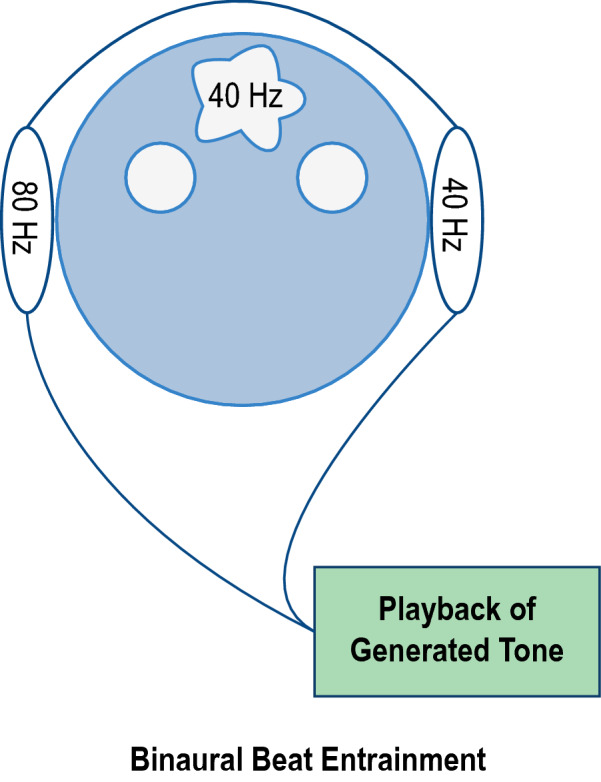
Example of binaural beat entrainment at 40 Hz

It is suggested that theta as well as Gamma oscillations contribute to episodic memory regulation [1]. As a result, interest in binaural beat entrainment has become more ubiquitous within recent decades. Binaural beat entrainment works by exposing an individual to two distinct and coherent tones operating at different frequencies. This results in a phenomenon, where an additional phantom frequency is interpreted by the cerebral cortex. The third frequency can be observed as the difference between the two coherent tones; hence, a left ear stimulation of 40 Hz and a right ear stimulation of 80 Hz would propagate the entrainment of a 40 Hz oscillation in the form of a binaural beat [2, 3]. An outline of the delivery mechanism and entrainment can be observed in Fig. 1.

In relation to human creativity, literature has examined its dependence on the neurotransmitter dopamine [4]. Moreover, there appears to be a relationship between creativity and the interactions between striatal and frontal dopaminergic neuronal pathways [5]. Meta-analyses reveal that, independent of anti-psychotic drugs, striatal D2 and D3 receptor density is, on average, 10–20% higher in those with schizophrenia [6–8]. Additionally, observations highlight that those with schizophrenia demonstrate an exaggerated creative ability in comparison to those without schizophrenia [9]. Interestingly, literature has also examined music (specifically the Mozart Effect) in pertinence to child creativity [10]. Further literature examining psychoacoustic stimulation in the form of a binaural beat finds evidence to suggest that binaural beat entrainment is able to regulate psychomotor skills as well as mood [11]. Moreover, this raises further questions as to whether psychoacoustic stimulation in other forms, such as a binaural beat, implicates human performance; more so in relation to cognition.

The understanding as to how areas of the nervous system interact with binaural beat entrainment is still not fully understood. However, some evidence suggests that the inferior colliculus and the reticular activation system facilitates in this perception [12–14]. Moreover, EEG (electroencephalogram) analysis has demonstrated that neuronal phase locking can be detected during binaural beat entrainment; where the observed neuronal action potentials are in synchronisation with a stimulus’ frequency [15]. Interestingly, the identification of particular waveforms that contribute to a given cognitive function may be achievable, especially with regards to memory [16].

A more recent multi-factorial meta-analysis has measured the efficacy of binaural beats in relation to anxiety, cognition as well as pain perception [17]. The factors were constituent of the duration, number of exposures and the frequency (Hz) of exposure. A key point during the analysis revealed that the effectiveness of a psychoacoustic stimulation cannot be affected by pink or white noise. Additionally, the findings also provide evidence to suggest that the exposure of a binaural beat before a task vs before and during a task may implicate binaural beat entrainment effectiveness, where a task in which an individual is exposed to a binaural beat before and during correlates with increased performance. The meta-analysis also provides evidence to suggest that a longer duration of psychoacoustic stimulation often results in exaggerated effects. In addition, further literature has examined working memory and emotion in relation to the Gamma 40 Hz binaural beat psychoacoustic stimulation [18]. The literature emphasises that a psychoacoustic stimulation lasting on average for 20 min enhances memory function whilst undergoing a recall task. Moreover, it may be advisable to increase the duration of time an individual is exposed to a binaural beat during an experiment. Though, criticism does identify that the measurements occurred only once for the two experiments, inferring a difficulty in accurately establishing long-term effects of such entrainment. Moreover, there appears to be a limited amount of literature examining a Gamma 40 Hz binaural beat in relation to cognitive benefits on a longer term basis.

Further interest in binaural beat stimulation stems from evidence suggesting that deregulation of brain wave activity can be observed within a multitude of disease pathologies [19–22], and hence potential treatments as well as mitigation strategies are of high value. With Alzheimer’s disease (AD) in particular, there is some evidence that Gamma 40 Hz photo-sensory entrainment is able to attenuate microglial load in genetically modified mice [23]. Criticism does highlight that mice models may not be representative of the reactions expected in humans. Preliminary studies applying entrainment frequencies demonstrate a varying degree of results. A particular study utilised photo-sensory Gamma 40 Hz stimulation with those suffering from AD as well as those with mild cognitive impairment (MCI) [24]. No significant changes in both groups were observed. Measurements occurred at the primary visual cortex, visual association cortex, posterior cingulate, precuneus, and lateral parietal cortex. Criticism does highlight that the particular study only took place for 11 days. This, when paired with an existing meta-analysis evaluating entrainment frequency duration benefits [18], may infer that longer entrainment may be necessary for the desired effects to be observed in humans. Additionally, an exploratory pilot study provides contradictory evidence in those psychoacoustically stimulated [25]. The experiment consisted of 18 participants suffering from AD, roughly distributed into groups dependent on disease severity: mild, moderate and severe. In pertinence to the mild and moderate groups, statistically significant improvements were observed (*p* < 0.05), conversely the inverse is true in that of the severe group. A difference to note with this study when compared to the previous, is the use of psychoacoustic stimulation rather than photo-sensory stimulation. Additionally, with positive results being observed, this may infer that psychoacoustic stimulation may be much more effective. Moreover, a lack of improvement noted within the severe group may infer that such stimulation is only effective as a mitigation or as a therapeutic therapy for those suffering with mild AD. Criticism does highlight the small sample size and nature of study being only preliminary.

It is worthy to mention here that this article is an extension of a previous paper presented at the 13th International Conference on Brain Informatics 2020 [26]. A justification for such an extension relates to the addition of extra results as well as a more elaborate discussion.

## Methods and experiments

### Entrainment frequency generation and software

Entrainment frequencies were generated utilising an internal piece of software in order to provide a full range of frequencies. Moreover, the software enabled the customisation of frequencies on a per channel basis. Additionally, the frequencies were generated in the .wav format due to the file-type being lossless in nature, further ensuring the preservation of frequencies used for psychoacoustic stimulation. The reasoning for this type of generation was, so that stereo headphones would be able to be leveraged in order to deliver independent frequencies to each of the participants ears. Finally, the internal piece software was developed in such a way that each participant would be able to be tracked and monitored over the course of the experimental phase, as well as all constituent results; moreover, allowing an analysis at a later date.

### Participants

Participants, who took part in the experimental phase of the research, were individuals who were able to lend their time for a duration of 4 weeks. The gathering of individuals was based entirely independent of gender, background or race. Social media advertisement was leveraged to enable the gathering of volunteers. In addition, participants were also over 18 years of age. Due to the exploratory nature of the research and the time limit, only 9 participants were able to be gathered. This has further caveats in terms of making truly conclusive statements, yet this amount of participants was deemed adequate given the nature of the research. As a result, participants were segregated into groups of three: leading to a split of 3, 3, 3; the 25 Hz, 40 Hz and the 100 Hz, respectively. For the duration of the experimental phase, participants were asked to continue their daily routines independent of the undergoing data collection phase. Additionally, all participants signed consent forms and confidentially guidelines were adhered to as per the BCS Code of Conduct. The consent forms ensured that the data acquisition, processing and statistical analysis were permissible. In relation to the developed internal software, participants were allocated unique identifiers to ensure that no personal data was required during any part of the experimental phase. Finally, the research was additionally approved by the Non-Invasive Human Ethics Committee. To elaborate further, the raw data collected was only stored for the duration of the experimentation phase. Upon conclusion of the research, all raw data were destroyed via a shredder. In relation to the internal software developed, we leveraged a SmarterASP.NET server, which utilised anti-virus protection and firewalls, following SSAE 18 SOC 2 Type 2 compliance. To conclude, all participants were freely able to remove themselves from the experimentation phase as well as all of their constituent data, given that the participant formally requested.

### Experiment

The experimental phase of the research spanned from the 12th of February 2020 up until a final session occurring on the 8th of March 2020. Participants would attend scheduled sessions between 10 a.m. and 10 p.m. every Wednesday as well as Sunday. An initial cognitive test would commence upon the arrival of a participant, under timed conditions. All relevant tests were recorded through the aid of a stopwatch. Moreover, upon conclusion of a timed test, a one second time unit would be removed from the final time; this aimed to reduce the impact of human error implicated by starting or stopping the stop watch. In order to assess the cognitive abilities of an individual, a custom model was proposed. The cognitive test aimed to assess individuals through the use of eight independent questions, each emphasising problem solving skills. An example of such a question would involve the ability of finding the next value within a given sequence of numbers. Further mathematical problems involved assessing an individuals understanding of operator precedence. Upon concluding the initial cognitive test for that particular session, a completion time was recorded and the stopwatch was reset. The participant would then be requested to return the paper, where a memory evaluation would commence. The memory evaluation was also a custom model which emphasised recall abilities. An example, would be to remember a sentence, solve a mathematical problem and then recall both to the researcher. Moreover, it is noted that the participants would never see these evaluations beforehand. Evaluations would be scored from 1 to 5, where 5 is a perfect score and 1 is a null answer. The completion of the first phase of session would then allow the exposure of a binaural beat. This would be delivered through a Sennheiser HD 400S headset with a sound pressure level (SPL) 120 dB (1 kHz/1 Vrms), frequency response 18–20,000 Hz (– 10 dB), total harmonic distortion (THD) < 0.5% (1kHz/100dB) with an acoustics closed sensitivity of 120 dB SPL @ 1 kHz, 1 V RMS (power). Given the recommendation from scientific literature, noting that a longer exposure to a binaural beat can contribute to a higher probability of positive results [17], participants were exposed to the corresponding binaural beat entrainment for 5 min. External factors were minimised, such that participants were asked to not interact with their mobile devices, potentially hindering the entrainment. Upon listening to the binaural beat, participants would be asked to repeat an additional cognitive test for that session, again, under timed conditions. Moreover, the same was true for a new memory test, performed with regard to the method already outlined. Finally, sessions would conclude upon completion of the final cognitive and memory tasks.

Additionally, a mood assessment would occur before undergoing psychoacoustic stimulation. The mood assessment was based on a pre-existing model of an already established Mood and Feelings Questionnaire (MFQ) [27, 28]. Moreover, mood assessments took place from the initial session, at the halfway point and on the concluding session: sessions 1, 4 and 8, respectively. With regard to mood scores, a lower score would infer an improvement in mood for that particular individual.

### Data processing

Data collected were converted to a percentage, with the intention of allowing for a more accurate comparison between the entrainment frequency groups. Additionally, MFQ scores remained in integer format due to a lower score correlating with a lower mood and the converse being true for higher scores. Upon the completion of result marking, the data were inputted into a Microsoft Excel work booklet. The data columns went as followed: session number, participant unique identifier, cognitive score before, cognitive before time, memory before score, cognitive after score, cognitive after time, memory score after and finally the MFQ score if applicable to the session. All cognitive, memory and mood overview scores were calculated as means over the duration of the 4-week experimentation phase.

### Statistical analysis

The statistical analyses were performed using the Graphpad Prism software (version 8.0). Cognitive as well as memory results were expressed in the form, mean ± SEM against the experimental condition; pre vs post. A homoscedastic Student’s *T*-Test was used to statically analyse the data for the duration of the experimental phase. Means were calculated for the frequency groups, which comprised the scores across the participants contributing to the observed results; additionally per the experimental condition. Individual results were plotted in grouped bar chart format with error bars indicating the SEM boundaries. Moreover, the same was true for the mean across frequency groups, at the population level.

Pearson’s product moment correlation coefficient (PMCC) test was leveraged during correlation analysis of MFQ scores for each individual participant. Additionally, the frequency group means were calculated based on the constituent participant scores for that group. Once performed, the same PMCC test was performed for each of the frequency groups. Additionally, for both individuals and frequency groups, data were plotted in scatter graph format. The correlation coefficient was plotted in line format showing the overall trend of the graphs.

## Results and discussion

The aim of this particular section is to present the data both at an abstracted population view as well as an individual view. A justification for such a format pertains to the nature of the study being exploratory; therefore only having 9 participants. Furthermore, presenting the data in this manner ensures that the observable contributions can easily be contextualised and understood. Moreover, it allows further discussion in terms of how much each individual is contributing towards an entire mean score observed at population level.

### Cognition

The intention of the cognitive assessment was to establish whether any significant changes would be observed with regard to the experimental condition (pre and post) for the duration of the experimentation phase. All values were given as mean ± SEM from week one until week four, due to literature suggesting that an increased duration of exposure would potentially correlate with improved entrainment results [17].

**Fig. 2:**
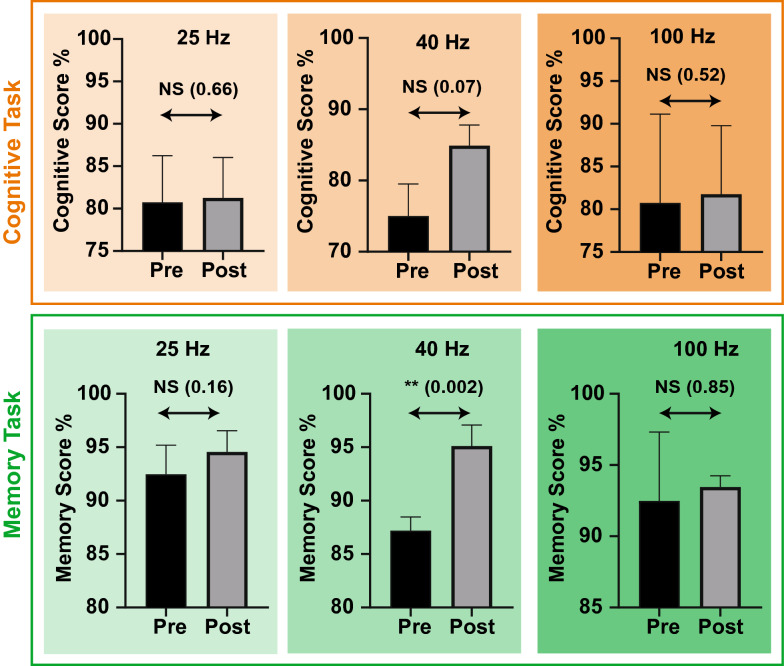
The cognitive task contextualises each frequency cohort (25 Hz, 40 Hz and 100 Hz) in relation to their respective cognitive scores (*y*-axis) with respect to the experimental condition both pre- and post-exposure to a 5-min-long binaural beat (top row). The memory task contextualises each frequency cohort (25 Hz, 40 Hz and 100 Hz) in relation to their respective memory scores (*y*-axis) with respect to the same experimental conditions (bottom row). Additionally, both cognitive scores and memory scores are given in percentages for ease of comparison. Bar graphs are constituent of the means for the duration of the 8 sessions over the course of the 4 weeks; error bars represent SEM. Moreover, cognitive and memory scores were statistically analysed using homoscedastic *T*-Tests. NS: statistically not significant, **: statistically significant

Abstracted at the population level, we observed no significant changes within the 25 Hz and 100 Hz, as seen per Fig. 2. However, statistical analysis revealed weak significance (*p* = 0.0761, $\alpha$ = 0.10) in relation to the 40 Hz frequency population, whereby an average increase in cognitive score elevated from 75 to 85%. To elaborate, the scores were analysed utilising a homoscedastic Student’s *T*-Test against the mean pre- and post-scores for the entire cohort constituting towards a particular frequency group. Criticism does highlight that all populations where constituent of small sample sizes, and therefore establishing true significance is not possible. However, such data may provide evidence to permit further experimentation; one in which the sample size is greater. Further evaluation notes a lower baseline score in relation to the Gamma 40 Hz group, when compared with 25 Hz and 100 Hz populations. This may highlight that the impact of entrainment may have greater impact on those with a lower baseline score, or that there may have been a higher tendency for scores to increase due to the naturally lower initial baseline. Moreover, it could be argued that had the 25 Hz and 100 Hz scores been lower during pre evaluations, then a similar observation could have been made. Irrespective of this, the results do appear consistent with the underlying roles of the Gamma frequency band, in which we observe higher functions persisting throughout regions of the brain [29]. Furthermore, it must be noted that it still remains unclear as to what underlying mechanism may be contributing towards the results observed within the Gamma 40 Hz frequency population. One method of aiding in the identification of such mechanisms pertains to the usage of an EEG to further analyse a participant during entrainment.

**Fig. 3:**
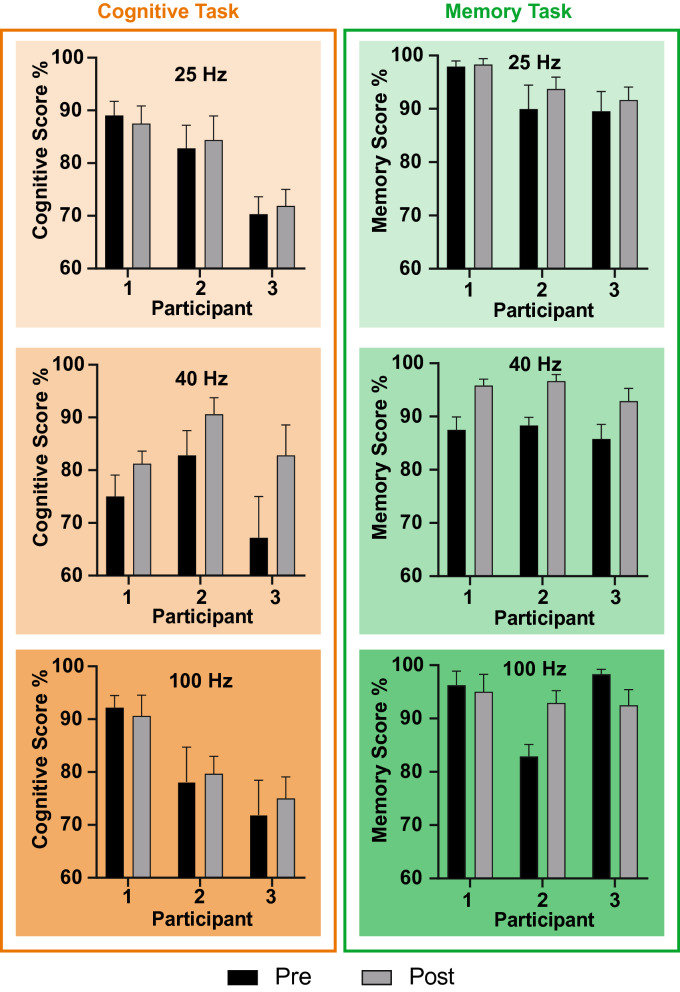
The graphs depict the individual cognitive and memory scores; each row separated by the constituent frequency groups (25 Hz, 40 Hz and 100 Hz) allocated as per the experimental phase. Additionally, the *y*-axis show the cognitive or memory score in percentage form. The *x*-axis displays the participants in numerical form; each corresponding to the correct participant across all three graphs. Moreover, it should be noted that these individual graphs each contribute to the overall task population graphs observed in Fig. 2. It should be noted that the 25 Hz cohort corresponds to participants 1–3 within the experimentation. The 40 Hz cohort corresponds to participants 7–9 within the experimentation. The 100 Hz corresponds to participants 4–6 within the experimentation

In relation to the individual data observed in Fig. 3, no significant changes between pre vs post were noted, except for participant nine. This reveals that for the majority of the population-wide data, significance may exist, yet those presumed benefits may not be observable amongst individuals contributing towards the overall trend. Moreover, due to the small groups, individuals have a 33% contribution towards the overall population observations. Therefore, the weak statistical significance noted within the 40 Hz cohort would have only existed due to the contribution from participant nine. Although, it should be said that population-wide benefits may only be confirmed through additional studies, where the sample size is far greater. Additionally, it raises further questions as to whether changes would still be observed during a more thorough quantitative analysis, using an electroencephalogram (EEG) for example, even if no qualitative differences are noted.

### Memory

The intention of this assessment was to establish, as well as acknowledge, whether exposure to any of the three frequency groups would result in performance changes with regard to memory. A population-wide analysis for both the 25 Hz and 100 Hz frequency groups revealed overlapping SEM, along with the *p* values 0.1616 and 0.8551, respectively. Furthermore, this leads to an observation that these results were insignificant.

The 40 Hz frequency cohort demonstrated a statistically significant increase from pre to post-scores over the duration of the experimentation (*α* = 0.05, *p* = 0.0027). Additionally, this is in line with existing evidence provided by a 2015 meta-analysis [30] as well as other literature on the topic of entrainment and memory improvements associated with both the Gamma and theta frequencies [1]. Moreover, the results observed add to the ever-growing research demonstrating memory improvements through exposure to binaural beat entrainment.

With regards to the individual data observed in Fig. 3, participants four and six demonstrated an overall mean decrease in memory on average when compared before and after. Interestingly, a statistically significant (*α* = 0.05) change in memory score, with a *p* value of 0.0469, is observed with participant six. Criticism highlights that the results observed are contradictory to existing literature which suggests that a 100 Hz Gamma entrainment frequency can improve memory [2]. A potential explanation to the results observed may relate to the small sample size. Moreover, an explanation of the high *p* value observed is noted if we consider the results that constitute towards the entire population Gamma 100 Hz frequency data; more so when considering that participant five demonstrates a statistically significant (*α* = 0.05, *p* = 0.04) result more consistent with the literature. It should also be noted that all individual contributions towards the 40 Hz cohort group were statistically significant with no overlapping SEM, which would further explain the observed population level increase, pre vs post, previously discussed.

### Mood

**Fig. 4:**
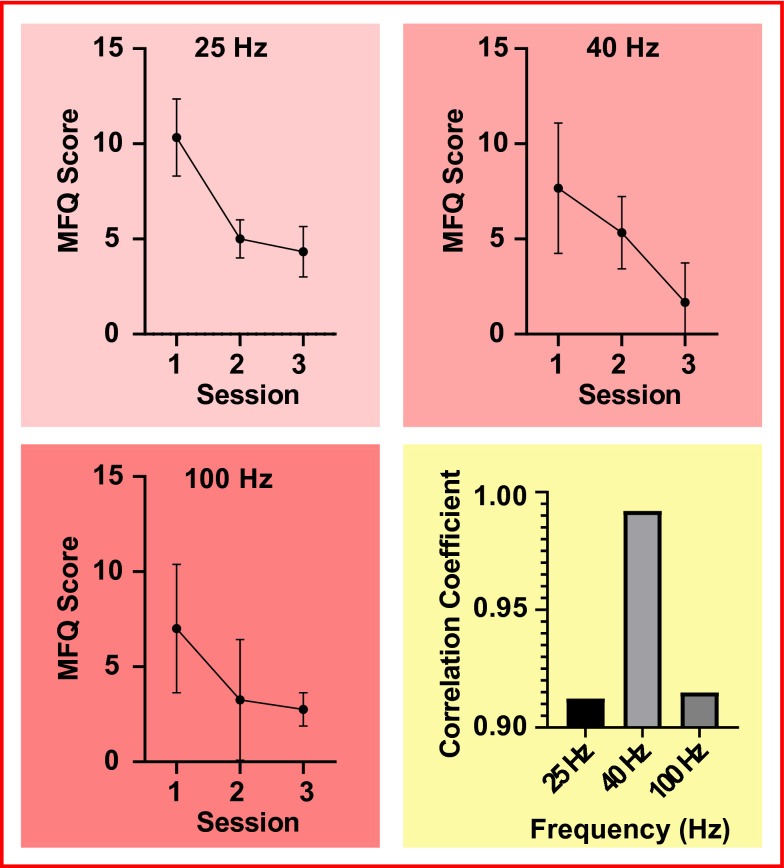
The three red shaded graphs depict the MFQ score (*y*-axis) changes over the sessions (*x*-axis) where mood questionnaires took place. In this instance, sessions 1, 2 and 3 refer to the study sessions 1, 4 and 8, respectively, where sampling occurred every 2 weeks during a 4 weeks period, including the start and end points of the study. Additionally, the final yellow shaded graph contextualises the correlation coefficient in pertinence to each frequency group, where the correlation efficient value is depicted by the *y*-axis and the frequency group is depicted by the *x*-axis

With regard to the mood portion of the experimentation, the general trend across all cohorts was a strong negative correlation between the MFQ score and the experimental condition (sessions), where a lower MFQ score indicated an improvement in overall mood. This, when regarded with literature suggesting Gamma frequency band attributes to mood improvements during entrainment [30], would highlight that the results observed are indeed consistent. Interestingly, referring to Fig. 4, the data suggest that the coefficient observed within the Gamma 40 Hz group (*R*^2^ = 0.9838) is stronger than the one observed in the Gamma 100 Hz (*R*^2^ = 0.8369) as well as the Gamma 25 Hz (*R*^2^ = 0.8322). This is consistent with the suggested evidence attributing improvements in mood with respect to the Gamma 40 Hz entrainment frequency in particular [18, 30]. This also highlights a potential that the Gamma 40 Hz entrainment frequency has a greater implication on overall mood when compared to the other frequency bands. Similarly, when the initial and final mean results are compared within the MFQ graphs that constitute Fig. 4, there is observed overlapping between the SEM values within the Gamma 100 Hz group. With this said, it could be inferred that the Gamma 100 Hz cohort mood improvements observed are likely to be statistically insignificant, even more so when considering the small sample size.

**Fig. 5:**
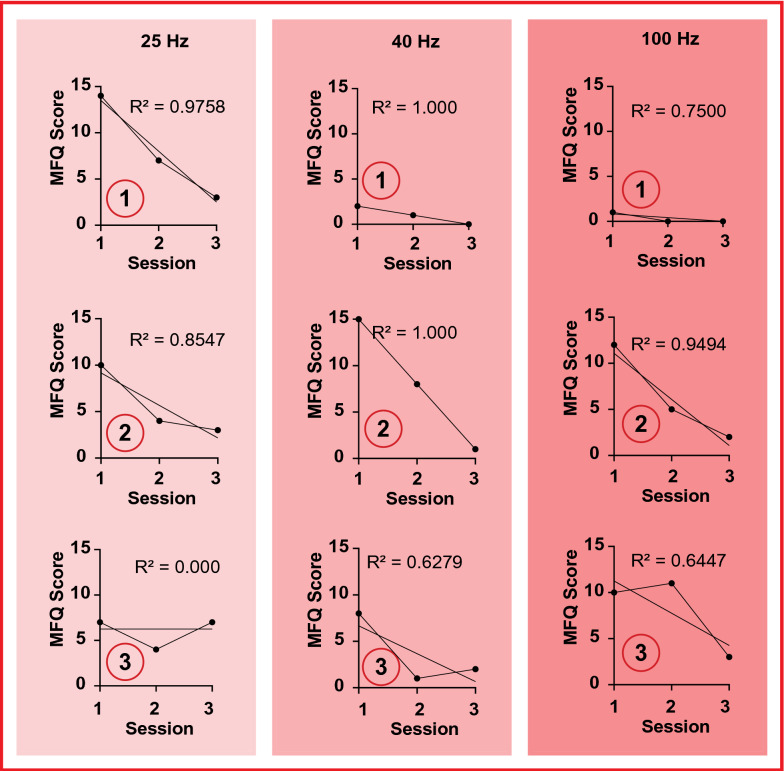
The graphs show the individual contributions to the overall trend observed in Fig. 4. The *x*-axis shows the sessions, normalised to the keep the results independent. It should therefore be noted that sessions 1, 2 and 3 in this case correspond to sessions 1, 4 and 8 within the actual experiment. The *y*-axis depicts the the MFQ score in integer format. Moreover, the *R*^2^ value is shown alongside the overall trend line for graph. Analogous to Fig. 3, the 25 Hz cohort corresponds to participants 1–3, the 40 Hz cohort corresponds to participants 7–9 and the 100 Hz corresponds to participants 4–6 within the experimentation

Individual correlation analysis reveals additional trends. When referring to Fig. 5, we observe a *R*^2^ value of 0 with participant three. This would indicate that there is little difference in mood when comparing directly with sessions 1 and 8 within the experimental phase. Although, for session 4 (session 2 within the MFQ plots), we observe an improvement in mood. A potential explanation for the trend observed could be a change in the participants life which had lead to an increased MFQ score. Further criticism highlights that, in these cases, the MFQ questionnaire might not be a viable solution for measuring changes in mood in pertinence to binaural beat stimulation. Similarly, this emphasises the requirement of a larger population size, since the contribution from a single individual hugely impacts the data observed, as per the overall population (Fig. 4).

### Potential application to chronic traumatic encephalopathy

Chronic traumatic encephalopathy (CTE) is a neurodegenerative disease which has been attributed to prolonged exposure of head injuries. It has therefore become highly regarded within the scientific consensus. This is especially true when considering a case study of a retired National Football League player [31]. CTE was first noted in 1928 by Harrison Martland. He described, what in the early 1920s was regarded as a condition known as punch drunk syndrome, a disease that was implicated by numerous repetitive head injuries (RHIs) in those that boxed [32]. The term dementia pugilistica was then eventually attributed to these neuropathological changes [33]. The name of progressive traumatic encephalopathy and later, CTE were preferred when activities other than boxing were attributed to the neuropathological changes consistent with the disease pathology [34]. Moreover, it is noted that the diagnosis of CTE in vivo within a living individual cannot be achieved. Furthermore, making the neuropathological symptoms difficult to diagnose and distinguish between other existing neurodegenerative disease [35].

The symptoms of CTE vary. Moreover, they can present themselves in a manner which is analogous to other neurodegenerative diseases; hence the high rate of misdiagnosis already noted. Some of the symptoms include memory impairment, mood related issues, executive dysfunction, language impairments and anger issues [36]. The same 2017 evaluation highlights, through a clinical top differential diagnosis, how often CTE may be mistaken for diseases such as Lewy body dementia, AD, frontotemporal dementia, vascular dementia and corticobasal degeneration. Furthermore, this additionally highlights how difficult epidemiological studies are to conduct with regard to CTE; more so when the prodromal stage of CTE may not exhibit one particular symptom. Literature has also examined the implications of RHI exposure with regard to CTE pathogenesis [37]. For the purposes of the evaluation, 36 individuals donated their brains for further analysis. At the time of their deaths 3 were asymptomatic, 22 presented mood and behavioural problems and 11 individuals presented cognitive impairment in some way. With this said, the same evaluation concluded that earlier RHI exposure correlates with mood issues and the latter correlates with cognitive issues. This led to a further conclusion that two forms of CTE exist: one emphasising mood issues, and the other emphasising cognitive problems. Additionally, due to more individuals presenting mood and behavioural issues, it could be assumed that this form of CTE is potentially more present amongst the population. Although this still needs further confirmation, as and when new diagnostic tools become readily available. A 2012 analysis of 85 brains found evidence to suggest that TBIs and repetitive mild traumatic brain injuries (mTBIs) trigger neuropathological changes characterised by the widespread deposition of hyper-phosphorylated tau protein (p-tau) in the form of neurofibrillary tangles [38]. Additionally, it could be argued that the p-tau accumulations throughout cortical layers 2 and 3 are specific to CTE. This is then further established with further literature distinguishing CTE from AD [39–41].

Neuropathological changes often observed with CTE have been noted in a high variety of sports such as football, boxing, wrestling, skiing and rugby. Additionally, these changes have also been noted in domestic abuse victims, head bangers as well as epileptics [35]. One factor that appears to be common across all cases of CTE, from the initially published case spanning from 1954 to 2013, are exposure to mTBIs [42]. Given that a relationship exists between mTBIs and CTE pathogenesis, prevalence of the disease may be severely underestimated. This is then further suggested by an epidemiological analysis highlighting that 42 million million worldwide suffer from mTBIs [43].

What still remains unclear is whether CTE exhibits any changes in wave power within regions of the brain; this is mostly due to a low amount of research regarding the topic. This is highly likely to be a result of the poor ability to accurately diagnose living individuals with the disease. However, there is some existing literature to suggest that deficits exist in pertinence to TBIs, an established precursor to CTE pathogenesis. A 2017 analysis noted deficits with regard to overall brain wave power implicated by individuals with mTBIs [44]. The analysis was constituent of 40 athletes, comprising 20 control individuals as well as 20 concussed individuals. Moreover, all individuals were analysed using an EEG. With regard to the results, evidence was presented that suggested that the concussed athletes demonstrated increased theta, delta and alpha power. It was also noted that there was a lowered beta power. Differences within the Gamma frequency band were not noted. However, further hidden frequency analysis revealed that from 1 to 40 Hz, major deficits at particular frequencies occurred. These hidden deficits were identified at the 1–2 Hz, 6–7 Hz, 8–10 Hz, 16–18 Hz, 24–29 Hz and finally the 34–36 Hz band. Interestingly, this highlights that although deficits were not noted within full bands, hidden deficits may still occur upon further analysis. This is then consistent with additional research on those with TBIs, finding disturbances and delays within the Gamma 40 Hz range [20].

Given the symptoms of CTE and that TBIs are a potential precursor to the disease pathology, along with a lacking amount of research associated with this disease in relation to entrainment. There may be a need to investigate the further implications of particular frequencies in mice models and those with TBIs, even more so in pertinence to the Gamma 40 Hz frequency entrainment.

## Conclusion and future work

To summarise, the work presented demonstrates that improvements in cognition, memory and mood can be observed in those undergoing binaural beat psychoacoustic stimulation at 40 Hz; with respect to the mean population results. Additionally, we observed improvements across the average mood for all frequency cohorts; with a greater *R*^2^ value presented by the 40 Hz cohort. Moreover, this work provides evidence to warrant further investigation; more so in pertinence to the underlying mechanisms that may be causing the observed results.

With regards to the limitations of this work, a greater sample size would have reduced the effects of various factors. A major factor is the weight of each individuals score contribution to the overall frequency cohort mean for each group. Further factors include the higher baseline scores observed within that of the 25 Hz and 100 Hz groups respectively; where these could have been normalised. Interestingly, this highlights that the lower score observed within the 40 Hz mean baseline may have been a deciding factor of the significance in relation to statistical analysis. Moreover, this may also highlight that those with lower baseline scores, such as those suffering with TBI’s, would benefit greatly. Additionally, an EEG analysis could have also been performed in parallel with the assessment; which may have also allowed the discussion pertaining to the expected responses from certain brain regions.
